# ^11^C‐Hydroxyephedrine PET/CT for preoperative surgical planning in large pheochromocytoma and paraganglioma

**DOI:** 10.1111/jne.70121

**Published:** 2025-11-30

**Authors:** Achyut Ram Vyakaranam, Olov Norlén, Alina Akural, Joakim Crona, Matilda Annebäck, Branislav Klimàcek, Peter Stålberg, Anders Sundin, Tobias Åkerström

**Affiliations:** ^1^ Department of Surgical Sciences—Molecular Imaging and Medical Physics Uppsala University Uppsala Sweden; ^2^ Department of Surgical Sciences—Endocrine Surgery Uppsala University Uppsala Sweden; ^3^ Department of Medical Sciences—Endocrine Tumour Biology Uppsala University Uppsala Sweden

**Keywords:** ^11^C‐hydroxyephedrine PET/CT, paraganglioma, pheochromocytoma

## Abstract

Early detection of metastases and timely surgical intervention play a crucial role in the management of neuroendocrine tumors. In large‐sized pheochromocytomas and sympathetic paragangliomas (PPGL), functional imaging with positron emission tomography (PET) is recommended, as it improves the detection of metastases, which may go undetected on conventional radiologic imaging. ^11^C‐hydroxyephedrine binds to the norepinephrine transporter receptor and is detected by PET/CT (HED‐PET/CT). It has previously demonstrated high accuracy in detecting primary and metastatic PPGL; however, its impact on preoperative staging is unclear. In this study, we retrospectively analyzed a selected cohort of 44 patients with large PPGL to evaluate whether HED‐PET/CT influences preoperative clinical decision‐making. All patients who underwent HED‐PET/CT at Uppsala University Hospital between 2004 and 2024 were screened for inclusion. In total, 44 patients with pheochromocytomas >5 cm and paragangliomas >4 cm were included. HED‐PET/CT results were compared with CT/MR findings, and a final consensus was reached on whether preoperative HED‐PET/CT would have altered clinical decision‐making. HED‐PET/CT identified previously undetected metastatic disease in three patients (6.8%), which had not been visualized on CT/MR. Additionally, two patients had discordant findings, where HED‐PET/CT revealed additional metastases. In one case, a liver metastasis was identified postoperatively with HED‐PET/CT, leading to a metastasectomy that could have potentially been avoided. These findings suggest that HED‐PET/CT is highly accurate in detecting metastases; however, its routine preoperative use may be limited and appears to provide significant clinical benefit only in selected patients.

## 1 INTRODUCTION

Pheochromocytomas (PCCs) are catecholamine‐secreting neuroendocrine tumors arising from chromaffin cells in the adrenal medulla, whereas sympathetic paragangliomas (PGLs) often arise along the sympathetic chain. Dysregulated catecholamine release in PPGL leads to clinical manifestations such as hypertension, headaches, palpitations, and sweating. These symptoms, although non‐specific, can be potentially life‐threatening.^1^ Coupled with the low incidence of the disease—approximately 0.6 PPGL cases per 100,000 persons annually^2^, ^3^—the diagnosis becomes both challenging and critical for reducing disease‐related mortality and morbidity.

Diagnosis of PPGL is primarily established biochemically, through detection of elevated levels of fractionated metanephrines or catecholamines in plasma or urine, followed by imaging studies, commonly Computed Tomography (CT), to localize and characterize the tumor.^4^, ^5^ Metastatic disease, defined by the presence of chromaffin cells in non‐chromaffin tissues, is evidence of metastases and is present in approximately 10%–15% of PCCs and up to 15%–35% of PGLs.^6^, ^7^ To allow accurate staging and detection of metastases, functional imaging is recommended, particularly for patients at high risk of metastasis, such as those with large tumors, rapid growth, or presence of pathogenic *SDHB* mutations.^5^, ^8^, ^9^ Clinical guidelines consider tumor size >5 cm for PCC and >4 cm for PGL as predictors of metastasis, and in such high‐risk cases, functional imaging studies are recommended.^5^, ^10^, ^11^

Positron emission tomography (PET) is widely used as a functional imaging tool, employing various radiotracers such as ^18^F‐FDG, ^68^Ga‐DOTA‐somatostatin analogues (SSA), 6‐[18F]‐L‐fluoro‐L‐3, 4‐dihydroxyphenylalanine (^18^F‐FDOPA), Meta‐[^18^F]fluorobenzylguanidine (^18^F‐MFBGP), and hydroxyephedrine (HED).^10^, ^12^ Owing to its higher sensitivity compared to CT/MR, functional imaging may improve the detection of metastatic disease and ultimately influence preoperative decisions. In patients with disseminated disease, this may lead to less aggressive surgery or modified surgical approaches, including resection of metastases, or the selection of open rather than minimally invasive surgery.^13^, ^14^

At our institution the norepinephrine analog‐based PET tracer, ^11^C‐hydroxyephedrine is used. This tracer demonstrates a specificity of 99%–100% and a sensitivity of 91%–96% in detecting PPGL.^12^, ^15^ Furthermore, HED‐PET/CT has shown high diagnostic value in post‐operative surveillance, with a sensitivity of 92% and specificity of 100%.^16^

A previous study demonstrated that functional imaging can change operative strategies and improve metastasis detection rates; however, the study primarily investigated preoperative ^18^F‐FDG PET.^17^ To further evaluate how functional imaging may alter clinical decisions we aimed to assess the utility of HED‐PET/CT as a preoperative imaging tool for large PPGL, focusing on its ability to detect metastatic disease and its potential impact on clinical decisions.

## 2 MATERIALS AND METHODS

This single‐center retrospective cohort study included patients treated at Uppsala University Hospital Uppsala, Sweden—a referral center for endocrine and neuroendocrine tumors. Our center routinely uses HED‐PET/CT as the first functional imaging modality for PPGL patients. In those with metastatic disease or equivocal findings, the institutional protocol, includes I‐131 MIBG scintigraphy in combination with ^68^Ga‐DOTATOC imaging for staging and assessment of therapeutic potentials.

**FIGURE 1:**
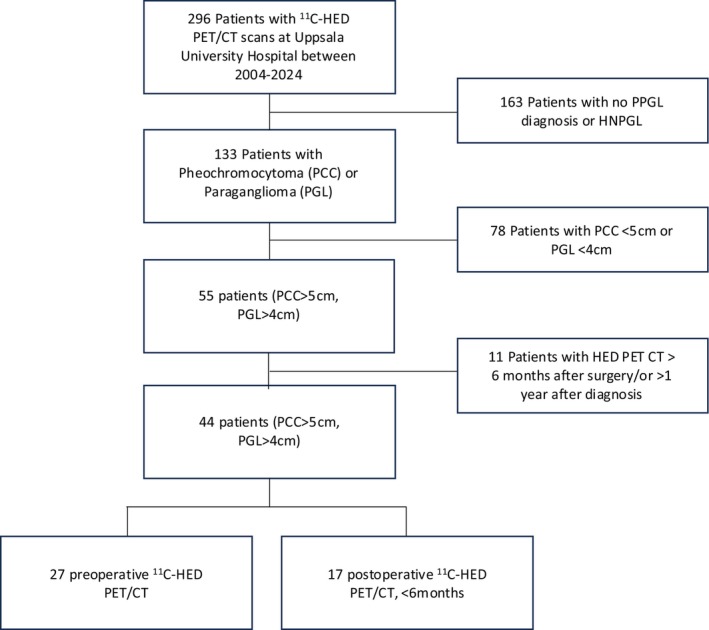
Flow chart of patient inclusion.

All patients who underwent HED‐PET/CT were screened for inclusion using data retrieved from the digital radiological information and picture archive and retrieval systems (RIS‐PACS) and patient charts between the dates 1st of January 2004 and 15th of December 2024. The study was approved by the Regional Ethics Board in Uppsala (Dnr 2012/422), and written informed consent from patients was obtained. Figure 1 outlines the patient inclusion and exclusion criteria. Exclusion criteria consisted of head and neck paragangliomas, patients who did not undergo surgery, and those with an HED PET/CT performed more than 1 year after diagnosis. Inclusion criteria were: (1) availability of both CT or MR and HED‐PET/CT imaging; (2) PCC >5 cm or a PGL >4 cm. Diagnosis of PPGL was established using standard clinical protocols including biochemical testing, functional imaging and histopathological examination. Patients with detectable metastasis within 6 months from surgery were classified as having synchronous metastatic disease.^18^

HED‐PET/CT was performed using a GE Discovery ST PET/CT scanner (General Electric Healthcare, Chicago, IL, USA). The PET scanner produces 47 slices with a 157 mm axial field of view (FOV) and 700 mm trans‐axial FOV. Patients received an intravenous injection of approximately 800 MBq of ^11^C‐HED, and 20 minutes post injection a static whole‐body examination scan was acquired from the base of the skull to the upper thighs. Physiological tracer uptake in salivary glands, myocardium, liver, spleen, pancreas and normal adrenal medulla was disregarded.

A non‐contrast‐enhanced, low‐dose CT was acquired prior to the PET scan for attenuation correction and anatomical correlations. PET images were reconstructed using Ordered Subset Expectation Maximization (OSEM) (2 iterations, 16 subsets) applying a 4.29 mm post‐processing filter. The PET data was normalized and corrected for dead time, random coincidences, physical decay, scatter and attenuation based on the low‐dose CT. Images were reconstructed into a 128 × 128 matrix, resulting in a spatial resolution of approximately 6.5 mm.

Retrospective data from imaging reports were retrieved from individual patient records. In separate reading sessions, metastatic lesions detected by CT/MR and by HED‐PET/CT, were recorded and compared to find concordant and discordant findings. To assess concordance between other imaging methods (MIBG‐scintigraphy, and ^68^Ga‐DOTATOC‐ PET/CT), we compared the overall lesion detection rate between these modalities. Only two patients performed ^18^F‐FDG PET/CT imaging, and these results were omitted. Only imaging performed within 6 months of the HED‐PET/CT were considered for analysis.

We then evaluated the clinical impact of HED‐PET/CT. Benign disease was determined not to be associated with a benefit, except in those with potentially multiple primary tumors. For patients with a preoperative HED‐PET/CT investigation, the decision from the Multidisciplinary cancer conference, based on the functional imaging results was considered. Additionally, a team of endocrine surgeons (PS, ON, MA, BK and TÅ) assessed the potential benefits of preoperative HED‐PET/CT on a case‐by‐case basis.

To avoid bias, we predefined clinical benefit as any HED‐PET/CT finding that led to or could have led to, a change in surgical planning. Such changes included: (1) less aggressive surgery; (2) local resection of liver metastasis; (3) resection of paraaortal lymph nodes; (4) conversion to an open procedure. Descriptive variables are presented as mean ± standard deviation, unless stated otherwise.

## 3 RESULTS

In total, 296 patients underwent HED‐PET/CT during the inclusion period. Of these 133 patients were diagnosed with PPGL. Among them, 55 had a PCC >5 cm or a PGL >4 cm. One patient did not undergo surgery, and the HED PET/CT was performed 2 years after diagnosis; therefore this patient was excluded. The remaining patients underwent primary tumor resection. Ten additional patients were excluded because their HED‐PET/CT was performed more than 6 months after surgery.

The final cohort consisted of 44 patients with PPGL who underwent HED‐PET/CT either preoperatively or within 6 months postoperatively (Figure 1; Table S1). This included 31 patients with pheochromocytoma >5 cm, 11 with abdominal paraganglioma, and 2 with thoracic paraganglioma >4 cm. The mean tumor size was 8.5 cm; the mean age at surgery was 55.3 years. The cohort included 23 male and 21 female patients (Table 1). Genetic testing was available for 24 of 44 patients using a panel of previously described genes involved in PPGL. Germline genetic testing showed pathogenic mutations in *SDHB* in three patients, one *SDHC* mutation, one *RET* mutation, one *NF1* mutation, and one with a variant of unknown significance in *VHL*. Metastatic disease was diagnosed in 16 patients (36.4%). Among these, 12 (75%) were classified as synchronous—defined as metastases detected preoperatively or within 6 months after surgery—and four as metachronous. Two cases were apparently benign, although follow‐up was limited to 6 months at data collection. We compared the lesion detection rate of HED‐PET/CT to MIBG‐scintigraphy and ^68^Ga‐DOTATOC‐PET/CT. This showed a higher detection rate than MIBG‐scintigraphy and a comparable rate to ^68^Ga‐DOTATOC‐PET/CT (Table 2). A comparison of previously published HED‐PET/CT results to other imaging modalities is provided in Table S2.

**TABLE 1:** Baseline patient characteristics.

Total cohort, *n*	44
Male	23 (52.3)
Female	21 (48.7)
Age, years, mean	55.3 (SD 17.0)
Genetic syndrome, *n* (%)	
*SDHB*	3 (6.8)
*NF1*	1 (2.2)
*SDHC*	1 (2.2)
*VHL (VUS)*	1 (2.2)
Pheochromocytoma (PCC), >5 cm	31 (70.5)
Paraganglioma (PGL), >4 cm	13 (29.5)
Tumor size, cm, median	8.45 (SD 4.0)
Malignant cases	16 (36.4)

*Note*: Values are number and (percent) unless otherwise stated.

Abbreviations: SD, standard deviation; VUS, variant of unknown significance.

**TABLE 2:** Comparison of lesion detection between different functional imaging modalities.

	MIBG‐scintigraphy	^68^Ga‐DOTATOC‐ PET/CT	HED‐PET/CT	^18^F‐FDG‐PET/CT
Patient 5	—	—	1	2
Patient 13	6	54	5	—
Patient 14	11	26	47	8
Patient 25	1	—	6	—
Patient 28	0	—	5	—
Patient 38	4	—	5	—
Patient 38 Exam 2	13	—	22	—
Patient 41	3	10	16	—
Patient 42	11	2	12	—
Patient 43	0	—	2	—
Patient 43 Exam 2	7	—	6	—
Total lesion count/patient	4.9	23	12.6	5
Lesions/patient MIBG vs. HED‐PET/CT	4.9	—	12.6	—
Lesions/patient ^68^Ga‐DOTATOC vs. HED‐PET/CT	—	23	20	—

### 3.1 Preoperative HED‐PET/CT findings

**FIGURE 2:**
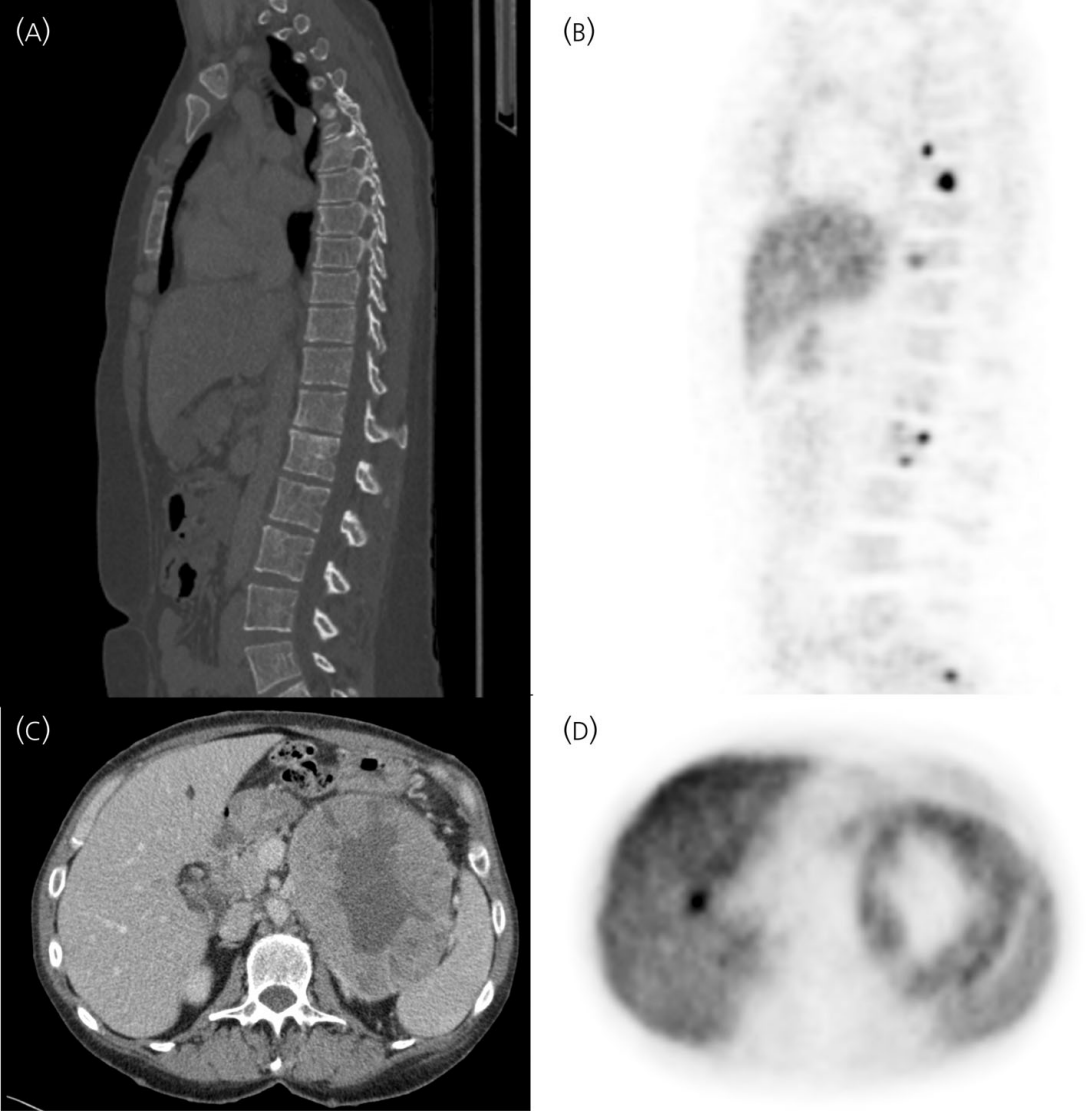
CT and HED‐PET/CT imaging results. (A) CT, in Patient 14, with no evidence of bone metastasis. (B) HED‐PET/CT, in Patient 14 showing multiple metastatic lesions. (C) In Patient 18, the preoperative CT scan shows a large primary pheochromocytoma but fails to identify the liver metastasis. (D) HED‐PET/CT scan show a liver metastasis with distinct tracer uptake.

Twenty‐seven patients underwent preoperative HED‐PET/CT (mean 36 days before surgery) and CT/MR. Among them, seven (26%) had metastatic disease. All patients had HED‐PET/CT positive lesions. In two patients (28.6%), preoperative CT failed to detect metastases (Table 3). This included bone metastasis in Patient 14 (Figure 2A,B) and both liver and bone metastases in Patient 18 (Figure 2C,D). In the remaining patients, findings from CT/MR and HED‐PET/CT were concordant. No changes in surgical decisions were made based on the HED‐PET/CT results.

**TABLE 3:** Discordant imaging results.

Id	CT/MR diagnosis	HED‐PET/CT diagnosis
Patient 14	No metastasis	Skeletal
Patient 18	Liver cysts	Liver, Skeletal
Patient 28	Thrombus	Thrombus, Skeletal
Patient 41	Lymph node	Lymph node, Lung, Skeletal
Patient 42	No metastasis	Liver

### 3.2 Postoperative HED‐PET/CT findings

**FIGURE 3:**
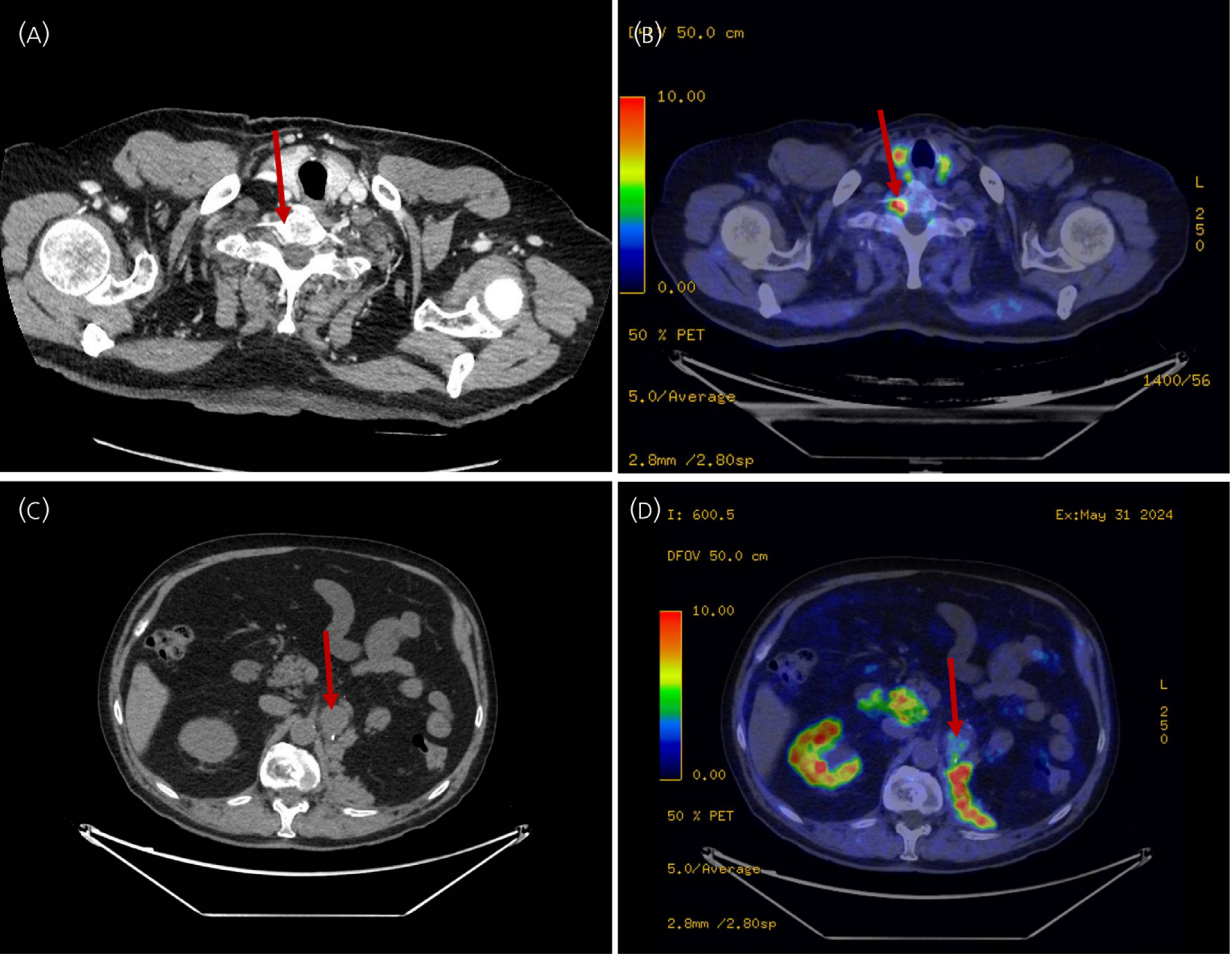
Discordant results in Patient 28 (A) CT of thorax with no sign of a C7 metastasis (arrow). (B) The C7 metastasis detectable with HED (arrow). Imaging results for *SDHC* patient (C) Local recurrence visible with CT (arrow). (D) Limited uptake of the HED‐PET/CT tracer in the recurrence.

**FIGURE 4:**
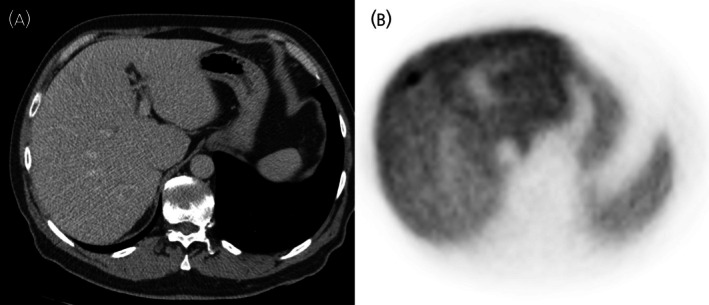
(A) In Patient 42, the liver metastasis on CT was undetectable. (B) Whereas, HED‐PET/CT showed distinct uptake in the metastasis.

Seventeen patients had undergone HED‐PET/CT within 6 months after surgery. Nine patients had metastatic disease, all showing HED‐PET/CT uptake. One patient with an SDHC germline mutation, exhibited both HED‐PET/CT avid and negative metastases/recurrences (Figure 3). Six patients were classified as having synchronous metastasis (<6 months after surgery) and three as metachronous (>6 months after surgery). Preoperative CT missed metastasis in two patients (Table 3). In patient 34, renal metastases were diagnosed intraoperatively; postoperative HED‐PET/CT revealed no additional metastases. Additionally, Patient 42 had a single liver metastasis detected postoperatively, which was ultimately treated by a second laparotomy with metastasectomy (Figure 4). Patient 41 had lymph node metastases detected by both CT and HED‐PET/CT; however, CT missed additional lung and bone metastases. In patient 28, CT correctly identified a tumor thrombus but missed a bone metastasis (Figure 3A,B). CT also suggested possible lung metastases in one patient, which were later diagnosed as benign nodules with HED‐PET/CT. In the remaining patients, CT/MR findings were concordant with HED‐PET/CT.

### 3.3 Overall clinical impact

HED‐PET/CT, performed either preoperatively or postoperatively, changed the diagnosis to metastatic disease in 3 of 44 patients (6.8%). When considering only those with malignant disease (*n* = 16), the staging was altered in 3 of 16 (18.8%). Furthermore, it detected additional metastatic lesions in two patients in the form of bone (*n* = 2) and lung (*n* = 1) metastases. In total 5 of 44 (11.4%) patients had additional metastatic sites detected using HED‐PET/CT.

To assess whether different staging based on HED‐PET/CT would have altered preoperative surgical planning, operative strategies were reviewed and discussed in a team of endocrine surgeons. Among patients with metastases who underwent HED‐PET/CT either preoperatively or within 6 months postoperatively, three required HED‐PET/CT for a diagnosis of metastatic disease and two had additional metastatic sites identified. All of these patients proceeded to primary tumor resection. All underwent open surgery and the surgical approach was not modified to be less aggressive owing to HED‐PET/CT findings. However, in Patient 42, a liver metastasis was detected postoperatively and it was concluded that, had HED‐PET/CT been performed preoperatively, the surgery would have been changed to avoid the need for a second laparotomy. Thus, despite the high lesion detection rate of HED‐PET/CT, only 1 of 44 (2.2%) patients would have undergone a different surgical approach if HED‐PET/CT had been performed preoperatively.

## 4 DISCUSSION

In this retrospective study, we compared HED‐PET/CT with CT/MR for detecting metastatic disease and evaluated its impact on preoperative decision making in patients with large PPGL. Compared with CT/MR, HED‐PET/CT detected additional metastatic lesions and altered clinical staging in 3 of 44 (6.8%), or 3 of 16 (18.8%) among those who ultimately developed metastasis. However, since most patients with a large primary tumor required surgery regardless of metastatic disease, HED‐PET/CT had limited influence on preoperative surgical decisions. In one patient (2.2%), a preoperative HED‐PET/CT would likely have changed the surgical plan. This patient had a single liver metastasis that was detected within 2 months of the primary surgery. This was subsequently treated with a metastasectomy, which could potentially have been performed at the first surgery had the lesion been identified preoperatively. In two other patients, additional lung, bone and liver metastases were detected by HED‐PET/CT. However, no change in the operative strategy was deemed necessary in these patients.

The standard diagnostic protocol for PPGL involves biochemical testing followed by CT imaging if biochemical markers are elevated.^5^, ^19^, ^20^ CT, owing to its high spatial resolution and sensitivity, is often sufficient to localize PPGLs.^21^, ^22^ Functional imaging, such as HED‐PET/CT, offers superior sensitivity and specificity, and increases detection rates of metastases.^12^, ^15^, ^16^ To date, no comparative analysis has been published evaluating it to other PET tracers. ^68^Ga‐DOTATATE‐PET/CT has replaced scintigraphy at several centers in the detection of primary tumors as well as metastasis. The fluorine‐18 labelled catecholamine precursor labelled with radioactive fluorine, ^18^F‐DOPA, although not available at our center, has been widely used to characterize PPGL preoperatively and its tracer uptake has been found to correlate with catecholamine secretion.^23^ The sensitivity of both ^18^F‐DOPA‐PET/CT and ^68^Ga‐DOTATATE‐PET/CT varies with tumor type.^24^, ^25^ In a cohort of 101 PPGL patients, ^18^F‐DOPA‐PET/CT demonstrated 93% sensitivity and 88% specificity.^26^ In another study comparing ^18^F‐DA, ^18^F‐FDG and ^18^F‐DOPA in 52 patients with PPGL, ^18^F‐DA was considered the preferred PET tracer, followed by ^18^F‐DOPA and ^18^F‐FDG.^27^ Furthermore, ^18^F‐DA has demonstrated better performance in both non‐metastatic and metastatic PPGL compared to SPECT, ^123^I‐MIBG and ^111^In‐pentreotide.^28^ However, how these modalities fare in large PPGL and how they affect the preoperative decisions remain largely unknown. Moreover, as noted above, direct comparison between HED‐PET/CT and these tracers is lacking.

Since 2004, Uppsala University Hospital, a Center of Excellence in Endocrine Oncology, has used HED‐PET/CT both for preoperative diagnostics and for postoperative surveillance of PPGL patients. Despite its diagnostic value, HED‐PET/CT is not widely available. One of the primary logistic challenges is the tracer's short half‐life (20 minutes), which is considerably shorter than that of other PET tracers labelled with ^68^Ga (68 min) and ^18^F (110 min). This requires specific handling, adding to the complexity and cost of this imaging modality.^12^ Despite its high sensitivity and specificity, the practical benefits of routine preoperative HED‐PET/CT remain uncertain, particularly when weighed against its logistical and economical challenges. Hence, it is primarily reserved for challenging cases, or for patients with a high risk of metastasis.

Surgical management of metastatic PPGL ranges from complete resection with curative intent to cytoreductive (debulking) procedures for symptom relief and biochemical control—even in advanced stages, as recommended by clinical guidelines.^5^, ^18^ Accurate staging may influence the surgical approach, including the choice of open versus laparoscopic surgery, the extent of resection and whether to perform metastasectomy. How HED‐PET/CT influences preoperative decisions has not yet been investigated, and the evidence supporting the use of other tracers is fairly limited. In a study by Nockel et al., ^18^F‐FDG PET/CT detected additional metastatic lesions compared to CT/MR in 15 of 93 (16%) patients evaluated preoperatively.^29^ This study included primary tumor resections, reoperations, and those with multiple primary tumors. In the subset undergoing primary tumor surgery, ^18^F‐FDG PET/CT was discordant in 7 of 66 patients (11%). These results are comparable to ours with HED‐PET/CT revealing additional metastatic lesions in 11.4% of patients. However, our study focused on a select group of PPGL patients with a high risk of metastasis. Nockel et al. also reported an association between the identification of additional lesions and selection of an open surgical approach. Laparoscopic surgery may limit intraoperative detection of paraaortic lymph nodes or non‐enlarged palpable nodes in the surgical field. In our cohort of PPGL patients we find that the practical implication of HED‐PET/CT prior to surgery is of limited value. However, many of our patients underwent open surgery. In centers that predominantly perform laparoscopic surgery, preoperative HED‐PET/CT could potentially help to identify otherwise undetected regional metastases. The impact of HED‐PET/CT in the postoperative surveillance of patients who fail to normalize their hormonal levels, and in those with evident biochemical recurrence, may be better, but requires further investigation. Additionally, in patients with borderline resectable tumors, HED‐PET/CT may provide valuable staging information. Further research in larger cohorts is required to clarify its role in such settings.

This study has several limitations. First, the relatively small sample size may limit the generalizability of the findings. As a single‐center retrospective analysis, the results are inherently influenced by our institutional protocols and may not fully reflect practices at other medical centers. To avoid bias in our results, we used predefined criteria for a change in clinical decisions. In addition, all patient cases were discussed by five surgeons, who reached an agreement. Furthermore, the study focused specifically on preoperative HED‐PET/CT on surgical planning limiting the applicability to other centers using other functional imaging modalities. Comparative studies between HED‐PET/CT and other tracers would be needed to fully understand the generalizability of our results. Despite this, the similar sensitivity and specificity between HED‐PET/CT and other PET tracers indicate that these results may in fact be applicable to other PET tracers. While inclusion criteria required a minimum follow‐up of 6 months, consistent with clinical guidelines for assessing surgical outcomes, this duration may still be insufficient in some cases. In addition, some surgeries were performed at other hospitals, introducing potential selection biases. As a tertiary referral center, our institution treats a disproportionate number of complex or advanced PPGLs, which may also affect generalizability. Finally, expert radiological interpretation may have influenced imaging findings for some patients in this study, and such expertise may not be uniformly available. Despite these limitations, our study raises an important clinical question: to what extent does functional imaging influence surgical planning and ultimately survival outcomes in patients with PPGL? Larger, multicenter studies will be essential to define the optimal role of preoperative functional imaging in this patient population and to determine which patients derive the greatest clinical benefit.

## 5 CONCLUSIONS

In this study, we evaluated the role of HED‐PET/CT in detecting metastatic disease and its impact on preoperative clinical decision‐making in patients with high‐risk PPGL. While HED‐PET/CT demonstrated high sensitivity in detecting metastatic lesions, it altered preoperative clinical management in only 2% of patients. These findings suggest that, although HED‐PET/CT provides valuable diagnostic accuracy, its routine use before surgery may have a limited impact on surgical planning.

## AUTHOR CONTRIBUTIONS

**Achyut Ram Vyakaranam:** Investigation; writing – original draft; writing – review and editing. **Olov Norlén:** Conceptualization; investigation; writing – review and editing. **Alina Akural:** Investigation; writing – review and editing; writing – original draft. **Joakim Crona:** Writing – original draft; writing – review and editing; methodology. **Matilda Annebäck:** Investigation; writing – review and editing. **Branislav Klimàcek:** Investigation; writing – review and editing. **Peter Stålberg:** Investigation; writing – review and editing. **Anders Sundin:** Methodology; conceptualization; writing – original draft; writing – review and editing. **Tobias Åkerström:** Conceptualization; investigation; funding acquisition; writing – original draft; writing – review and editing; methodology; supervision.

## CONFLICT OF INTEREST STATEMENT

The authors declare no conflicts of interest.

## ETHICS STATEMENT

The study was conducted in accordance with the Declaration of Helsinki, and approved by the regional Ethics committee in Uppsala (Dnr 2012/422). Informed consent was obtained from all subjects involved in the study.

## Supporting information


**Table S1.** Patient characteristics.

**Table S2.** Previously published results on HED‐PET/CT, and comparison to other imaging modalities.

## Data Availability

The data that support the findings of this study are available on request from the corresponding author. The data are not publicly available due to privacy or ethical restrictions.
